# Circulating miRNAs as Biomarkers for Prostate Cancer Diagnosis in Subjects with Benign Prostatic Hyperplasia

**DOI:** 10.1155/2020/5873056

**Published:** 2020-05-08

**Authors:** Wei Jin, Xiang Fei, Xia Wang, Fangjie Chen, Yan Song

**Affiliations:** ^1^Department of Urology, Shengjing Hospital of China Medical University, Shenyang, Liaoning, China; ^2^Department of Medical Genetics, School of Life Sciences, China Medical University, Shenyang, Liaoning, China

## Abstract

Body fluids often contain freely circulating nucleic acids, many of which can be exploited as noninvasive tools for the diagnosis of cancer as well as for clinical prognostication. Identifying microRNAs (miRNAs) in subjects' blood with various malignancies means that they can serve as novel biomarkers for prostate cancer (PCa) diagnosis. This study analyzed serum-circulating miRNAs as a noninvasive biomarker in subjects with PCa and subjects with benign prostatic hyperplasia (BPH). In total, 31 PCa subjects and 31 BPH subjects were included, with the BPH group serving as the control group. RT-qPCR was used to quantify the levels of 10 miRNAs, which included miR-18a, miR-34a, miR-106b, miR-183, miR-200a, miR-301a, miR-141, miR-182, miR-200b, and miR-375 in serum. Statistical tests were used to assess the relationship between the levels of miRNAs and the clinicopathological data. A significant increase was observed in the relative expression ratios of miR-141, miR-182, miR-200b, and miR-375 (1.89-, 2.09-, 2.41-, and 2.27-folds, respectively) in the PCa group when compared to the BPH group. Based on the receiver operating characteristic (ROC) analysis, the largest area under the curve (AUC), 0.923, was associated with the miR-200b group, indicating effective diagnostic properties for this biomarker. A correlation was observed between total prostate-specific antigen (TPSA) and the relative levels of miR-141, miR-182, miR-200b, and miR-375. The Gleason score and the miR-200b expression level were also correlated. These results are consistent with previous studies regarding the possibility of differentiating between PCa subjects and healthy controls based on the detection of miRNA. The findings attest to a distinctive expression profile of miRNA that is detectable in the blood of PCa subjects, thereby confirming the role of miRNAs as diagnostic biomarkers for PCa.

## 1. Introduction

As the fifth most common cause of cancer-related deaths in men and the second most common malignancy, 1.3 million new cases of prostate cancer (PCa) were diagnosed in 2018, and 358,989 deaths were recorded globally in the same year [1]. While PCa incidence in China is not as high as that in Western countries, it has increased in recent years [2–4]. PCa is marked by its heterogeneity; thus, some PCa patients experience severe clinical symptoms or even death, while other PCa subjects do not [1]. It is estimated that around 16% of men will receive a PCa diagnosis at some point in their lives [5].

Serum total prostate-specific antigen (TPSA) and digital rectal examination (DRE) are commonly used screening tools for PCa. The former is a biomarker that is regularly used for PCa detection. A key limitation of serum TPSA is its low specificity and sensitivity to PCa [6]. Devising an effective treatment pathway for a PCa patient depends on several factors, including the patient's serum TPSA levels, Gleason score, and clinical stage; however, the issue is complex, as in many cases, subjects with similar clinical conditions experience contrasting outcomes [7]. In the case of DRE, interexaminer variability is a fundamental limitation, and the screening tool can only assess peripheral zone tumors. Nevertheless, given its cost-effectiveness, availability, and ability to detect tumors in 14% of PCa subjects, DRE is a key screening initiative [8, 9]. Importantly, as indicated by several PCa screening trials, including the European Randomized Study of Screening for Prostate Cancer (ERSPC) and the Prostate, Lung, Colorectal, and Ovarian (PLCO) cancer screening trial, the diagnosis rate for PCa subjects has increased; however, the mortality rate has not improved [10–13]. In light of this fact, viable screening and prognostic biomarkers for PCa patient management must be identified [14].

As noncoding RNA molecules that each contain around 22 nucleotides, microRNAs (miRNAs) inhibit gene expression at the level of translation by targeting mRNA molecules directly [7]. It is possible for miRNAs to modulate the level of translation of around 30–50% of human genes, and they play a regulatory role with respect to molecular signaling pathways in cells. The literature indicates that miRNAs circulate in biological fluids (e.g., the blood) with a high level of stability, which broadens their possible function as both fluid-circulating and tumor-specific biomarkers [15]. In 2007 and 2008, the first studies addressing PCa-altered miRNAs were conducted, with the main aim of detecting miRNA profiles associated with predictive, prognostic, and diagnostic capabilities [16, 17]. In recent years, researchers have identified a range of miRNA expression profiles in tumors of the prostate, all of which exhibit extensive overall deregulation of miRNAs [17, 18]. Studies indicate that many serum miRNAs are expressed differently in the context of PCa, where a significant proportion are oncomirs, displaying overexpression in PCa, promoting tumorigenesis, and negatively regulating specific tumor suppressor genes [19, 20]. Therefore, these stable, cancer-specific biomarkers have been identified as having considerable potential in the diagnosis, prognosis, and prediction of treatment responses to PCa.

Given the promise associated with miRNAs as cancer biomarkers, a range of research projects has been undertaken to identify the most relevant miRNAs implicated in PCa biology and to establish a PCa-specific miRNA expression profile [21, 22]. Song et al.'s meta-analysis identified 10 upregulated miRNAs (miR-18a, miR-34a, miR-106b, miR-183, miR-200a, miR-301a, miR-141, miR-182, miR-200b, and miR-375), each of which was associated with effective predictive and diagnostic potential in differentiating between PCa subjects and benign prostatic hyperplasia (BPH) controls [23]. This study's objective was to identify upregulated miRNAs in subjects for the routine detection of PCa. The 10 aforementioned miRNAs were analyzed in the sera of both PCa and BPH subjects. Additionally, an analysis was undertaken of the relationship among circulating miRNA levels and TPSA and the Gleason score.

## 2. Materials and Methods

### 2.1. Study Design and Hemolysis Assessment

The Research Ethics Committee at Shengjing Hospital of China Medical University provided ethical approval for this research project. Voluntary and written informed consent was sought from and documented for every patient. Between January 2018 and July 2019, 62 BPH subjects with suspected PCa were recruited into the study. DRE and PSA tests were used to facilitate early PCa detection, and the dissection of the prostate specimens took place during the routine collection of fragments for histopathological evaluation. In total, 31 subjects eventually received a PCa diagnosis, while the remaining 31 subjects were diagnosed as having BPH. Intravenous infusion, which relied on needles and disposable BD Vacutainer® tubes containing 6% ethylenediaminetetraacetic acid (EDTA), was employed to obtain peripheral blood samples from all subjects. Centrifuging of the blood samples at 700 g for a 10-minute period took place within 120 minutes of the samples being placed on ice for processing. To avoid cellular contamination, circulating RNA enrichment was undertaken based on the procedure described by Duttagupta et al. [24]. Blood plasma was centrifuged at 2,000 g for a 10-minute period at 4°C. After the centrifuging process, the storage of the cell-free plasma took place at −80°C until it was used for miRNA detection.

### 2.2. RNA Extraction and Reverse Transcription

Several changes were made to the manufacturer's recommended protocol when using the miRNeasy Mini Kit (Qiagen, Hilden, Germany) to extract the total circulating RNA. Specifically, a 1 mL plasma sample was separated into 5 tubes, each with 200 *μ*L plasma. After 1 mL TRIzol™ reagent (Thermo Fisher Scientific) was used to treat each tube, the tubes were vortexed for 60 seconds and subjected to incubation at room temperature for a 5-minute period. After the incubation, 200 *μ*L chloroform was used to treat the mixture, and it was vortexed for 15 seconds. After incubating the solution at room temperature for a 180-second period, centrifugation at 12,000 g took place for 15 minutes at 4°C. The supernatant was transferred to a fresh tube, and homogenization of the supernatant occurred with 1.25 volumes of 100% ethanol. In total, 700 *μ*L of the solution was placed into a binding column with a collection tube, after which it was centrifuged at 8,000 g for 15 seconds. This process was repeated 12 times, eliminating the flow-through after each iteration. Following the saturation of the column, the column was washed, and 25 *μ*L RNase-free water was used to elute the sample. Quantification of the samples was conducted using a NanoDrop 2000 spectrophotometer (Thermo Fisher Scientific, Wilmington, DE, USA).

### 2.3. miRNA Detection

Next, 5 ng circulating RNA and the TaqMan miRNA Reverse Transcription kit (Applied Biosystems, Foster City, CA, USA), applied in accordance with the manufacturer's recommendations, were used to perform the expression patterns of the miRNAs. The elution of every reaction involved a 1 : 4 ratio, and the reactions contained 5.5 *μ*L of TaqMan 2x Universal PCR Master Mix (Applied Biosystems, WA, UK), 0.45 *μ*L of a miRNA-specific TaqMan probe, and 7 *μ*L cDNA of the diluted RT reaction.  Table 1 provides an overview of the primers used in the research project, and the RNU6 gene was utilized as a control gene. The mathematical model of 2-*ΔΔ*CT was applied to acquire the relative expression data.

### 2.4. Statistical Analysis

SPSS 25.0 (IBM-SPSS Inc., Chicago, IL, USA) was used to perform the study's statistical analysis, along with GraphPad Prism 7.0 software for Windows 10 (GraphPad Software Inc., CA, USA). For the data analysis, Spearman's rank correlation and the Mann-Whitney *U* test were applied. The receiver operating characteristic (ROC) curve was employed for the purpose of evaluating the specificity, sensitivity, and cut-off points of each marker and diagnostic test and to facilitate a comparison between the PCa subjects and the BPH subjects. Logistic regression was used to combine the biomarkers, and *P* < 0.05 was considered a statistically significant result.

## 3. Results

**Figure 1:**
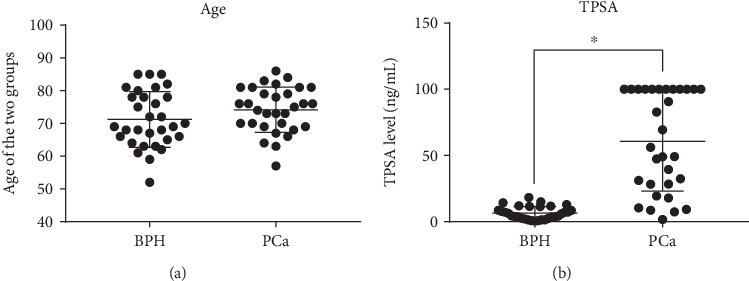
The ages and TPSA levels in the PCa and BPH groups: (a) age; (b) TPSA level. ^∗^*P* < 0.05; *n* = 31.

**Figure 2:**
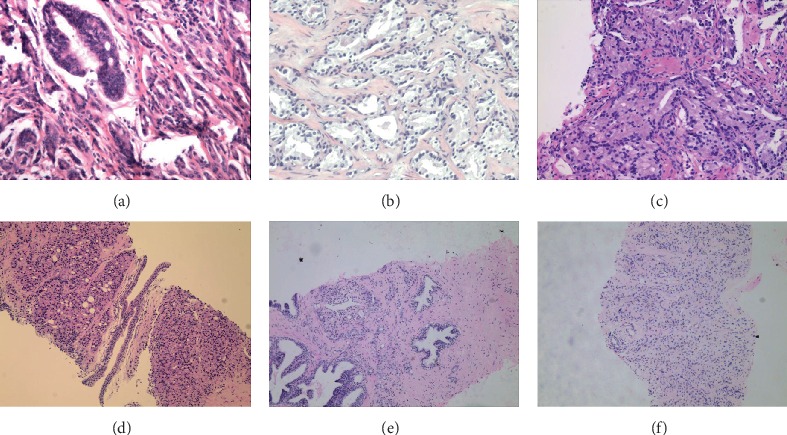
Typical images seen in a pathological examination. (a) A subject with a Gleason score of 5, (b) a subject with a Gleason score of 6, (c) a subject with a Gleason score of 7, (d) a subject with a Gleason score of 8, (e) a subject with a Gleason score of 9, and (f) a subject with a Gleason score of 10.

The clinicopathological characterization of the included subjects is outlined in Table 2, where the mean age, age range, mean total PSA, and median Gleason grade were 74.2 years, 57–86 years, 60.63 ng/mL, and 8, respectively. For the BPH group, the mean age and age range were 71.2 years and 52–85, respectively, while the mean total PSA was 6.57 ng/mL (Table 2). No statistically significant difference was detected in terms of age; thus, age was not considered the factor that accounted for the disparities between the PCa and BPH subjects (Figure 1(a)). The TPSA level in the PCa group (*P* < 0.05) was significantly higher than that in the BPH group (Figure 1(b)). The typical Gleason score for a pathological examination is outlined in Figure 2.

**Figure 3:**
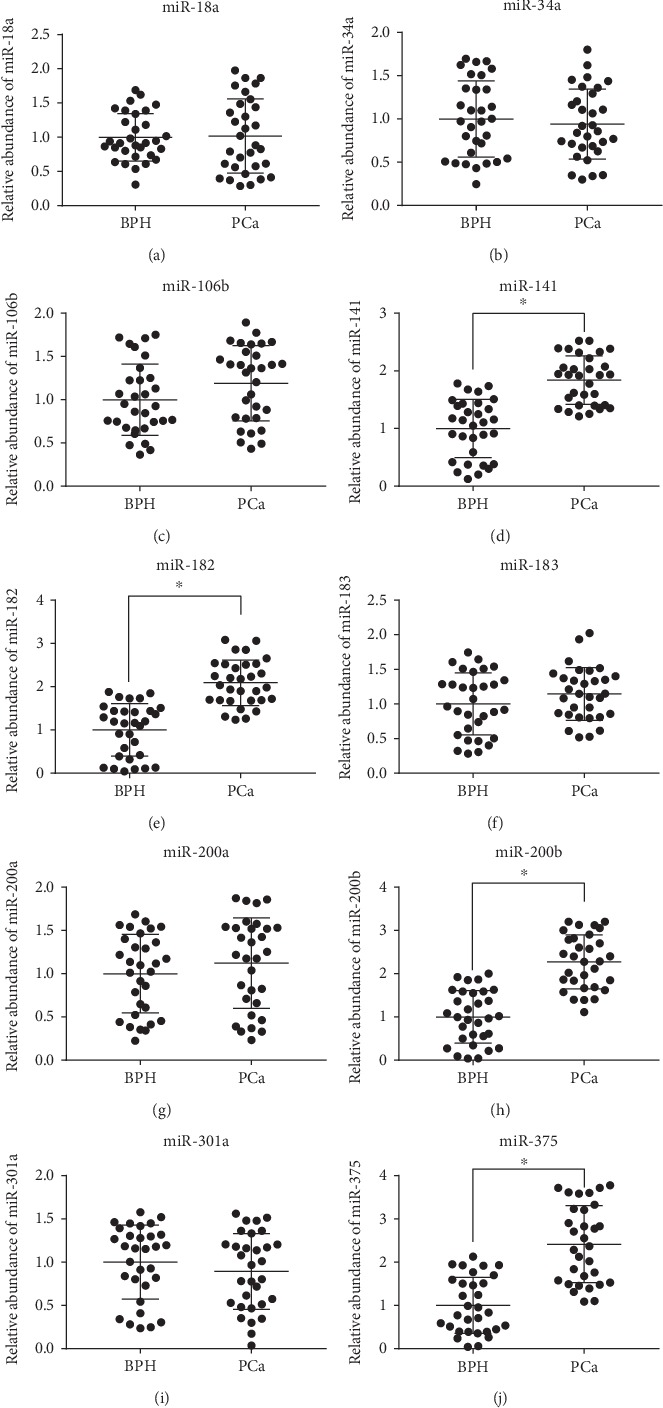
The relative miRNA levels in the PCa and BPH groups: (a) miR-18a, (b) miR-34a, (c) miR-106b, (d) miR-141, (e) miR-182, (f) miR-183, (g) miR-200a, (h) miR-200b, (i) miR-301a, and (j) miR-375. ^∗^*P* < 0.05; *n* = 31.

**Figure 4:**
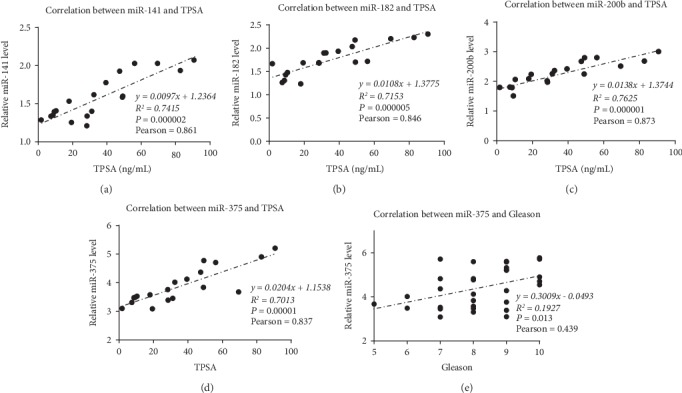
Correlations between the miRNAs and the TPSA level or Gleason score in the PCa subjects. (a) The correlation between miR-141 and TPSA, (b) the correlation between miR-182 and TPSA, (c) the correlation between miR-200b and TPSA, (d) the correlation between miR-375 and TPSA, and (e) the correlation between miR-375 and the Gleason score. In our hospital, TPSA levels of higher than 100 ng/mL were marked as >100 rather than an extract number; thus, the subjects with TPSA levels greater than 100 ng/mL were excluded from the correlation analysis between the miRNAs and the TPSA level.

Normalization of the expression ratios of specific miRNAs from the serum samples of the PCa and BPH subjects was undertaken, with RNU6 used as a reference gene. In this case, miR-141, miR-182, miR-200b, and miR-375 (1.89-, 2.09-, 2.41-, and 2.27-folds, respectively) were statistically higher (*P* < 0.05) in the PCa subjects than in the BPH subjects (Figure 3). No significant difference was observed in the expression of the other six miRNAs (miR-18a, miR-34a, miR-106b, miR-183, miR-200a, and miR-301a) between the two groups of subjects. The correlation analysis within the PCa group indicated that miR-141 (Figure 4(a)), miR-200b (Figure 4(b)), miR-182 (Figure 4(c)), and miR-375 (Figure 4(d)) were correlated with TPSA. However, we only detected a correlation between miR-375 and the Gleason score (Figure 4(e)); we did not detect any correlation between the Gleason score and miR-141, miR-200b, or miR-182.

**Figure 5:**
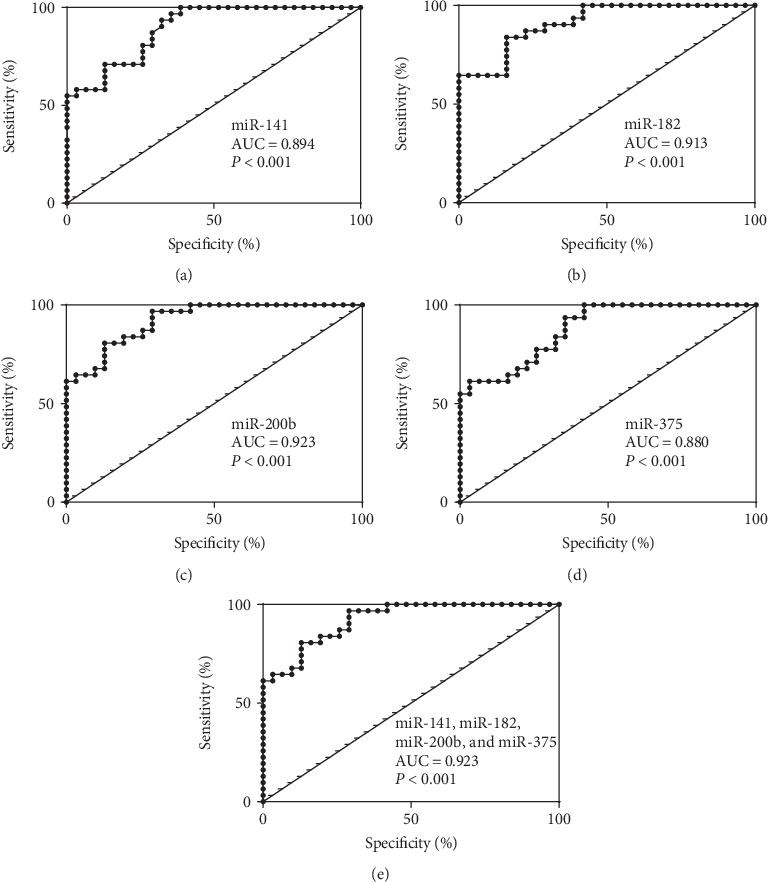
The ROC curve analysis of the miRNA levels in the PCa and BPH groups. (a) The ROC curve of miR-141, (b) the ROC curve of miR-182, (c) the ROC curve of miR-200b, (d) the ROC curve of miR-375, and (e) the combination of four miRNAs (miR-141, miR-182, miR-200b, and miR-375) in the PCa and BPH groups.

To assess the capacity of miRNAs to permit differentiation between BPH and PCa serum samples, an ROC analysis was undertaken (Table 3 and Figure 5). The largest value for the area under the curve (AUC) was associated with miR-200b (0.923, 95% CI between 0.8618 and 0.9842), and miR-141, miR-182, and miR-375 displayed high AUC values of 0.894 (95% CI between 0.8197 and 0.969), 0.913 (95% CI between 0.8459 and 0.9793), and 0.880 (95% CI between 0.8001 and 0.9605), respectively. The ROC analysis of the combination of the four circulating miRNAs indicated no significant improvements in the AUC values (AUC = 0.923, 95% CI between 0.8620 and 0.9840) (Figure 5).

## 4. Discussion

Although the analysis of circulating nucleic acids is associated with a range of technical limitations, these molecules have been quantified as robust biomarkers for cancer diagnosis [25]. A key disadvantage of cell-free miRNA assessments is their low abundance [26], and miRNAs are associated with intracellular mRNA contamination, degradation, and low stability. Nevertheless, changes in circulating miRNAs are indicative of aberrant cancer immunity, cell growth, stromal interaction, and proliferation, meaning that these miRNAs can be used for diagnosis and prognostication [25–27]. In this study, 10 PCa-specific miRNAs were quantified in PCa subjects' serum samples, and miR-141, miR-182, miR-200b, and miR-375 were higher in the PCa subjects compared to the BPH subjects. In line with results from previous studies [21, 28], overexpression of miR-141, miR-182, miR-200b, and miR-375 was detected in the PCa serum samples more so than in the BPH subjects, which relied on the use of real-time qPCR analysis.

The results indicate that, in the PCa subjects, the miR-141 mean expression levels were substantially higher. This implies that miRNAs could be leveraged in clinical settings to determine which subjects are at elevated risk of PCa due to higher PSA. In a previous study, the researchers noted that miR-141 could identify PCa subjects with an AUC amounting to 0.9, but each of the PCa subjects included (*n* = 25) suffered from metastatic disease [15]. The mean expression levels of miR-141 may increase as the disease spreads to other organs and moves through the locally advanced and metastatic phases [29]. With this in mind, it is clear that considerable variability exists in the results, particularly in terms of the specific blood products, extraction methods, and endogenous controls used.

The present study verified that miR-182 was overexpressed to a significant degree in the PCa subjects compared to the BPH subjects, thereby lending additional weight to its oncogenic role in prostate carcinogenesis [30, 31]. It has been reported in the literature that miR-182 expression levels alone are sufficient for differentiating between tumorous and nontumorous prostate tissue, and specificity, PPV, and AUC values of 100%, 99.55%, and 0.81, respectively, were achieved [32]. These results are favorable compared to those of an earlier study, which reported that an AUC of 0.70 (95% CI: 0.62–0.79) was achieved, and where 76 matched PCa and adjacent healthy tissues were considered [31]. Despite the fact that the inadequate sensitivity of a single circulating miR-182 analysis may be low, thereby contributing to poor results, it may represent an effective adjunct to other available tests. As such, it could increase the specificity of serum TPSA testing, possibly lowering the requirement for a needless biopsy [32].

In the case of miR-200b, the expression level differed between the control group and the PCa subjects. Regulatory impacts come from the members of the miR-200b family and affect genes implicated in the epithelial-mesenchymal transition [33]. This study's results are consistent with previous research [33], where lower levels of circulating miR-200b were quantified in the serum samples of the controls compared to the PCa subjects. Compared to the control group, subjects suffering from PCa displayed a threefold downregulation in their miR-200b expression. Elsewhere in the literature, researchers have found that the miR-200b expression tends to fall in PCa tissue samples and cell lines [33, 34].

miR-375 is primarily considered a tumor suppressor, specifically in head and neck, pancreatic, hepatocellular, and gastric cancers [35–37]. Some researchers have found that forced miR-375 expression in gastric cancer results in higher apoptosis and lower cell viability *in vitro* [38, 39]. Additionally, Haldrup et al. reported on a 14.66-fold change in the level of serum miR-375 in PCa subjects, as well as relatively low diagnostic properties (AUC = 0.650) [21]. In the present study, the results were not the same in terms of AUC and fold-change expression, indicating a lower level of differentiation between the BPH and PCa subjects. However, the diagnostic values in this study were higher than those reported elsewhere in the literature, with an expression level of 2.27 and an AUC value of 0.880. It is worth highlighting, though, that miR-375-altered levels are primarily based on microarray or validation platform analysis attempts to differentiate between tumor subgroups based on miRNA expression. Biological clues about the role played by miR-375 in cancer are provided in very few cases [36, 40]. Brase et al. [41] detected a correlation between the Gleason score and miR-375, which partly reflects this study's results (specifically, this study found a significant correlation between miR-375 and the Gleason score at *P* = 0.013). In line with previous studies [21, 42], miR-375 levels were directly correlated. Again, partially consistent with this study, the researchers reported that the miRNA-375 expression is increased in PCa subjects, and its release into the blood is a sign of PCa [21, 42].

Given that PCa screening must be improved [34], this study's finding that the combined use of miR-141, miR-182, miR-200b, and miR-375 as a sensitive panel of biomarkers with an AUC value of 0.923, which aided in differentiating between the PCa and BPH subjects, is significant. These results are indicative of the potential role of circulating miRNAs as efficient biomarkers of complex health conditions and are consistent with previous studies that explored circulating miRNAs in PCa diagnosis, as well as in terms of exploiting a collection of miRNAs to aid in the diagnosis of PCa. In light of the sensitivity of the technique, as well as the limited number of false negatives, quantifying a panel of circulating miRNAs clearly represents a potentially valuable way to limit the number of biopsies, to guide PCa treatments in an evidence-based way, and to permit risk stratification for active surveillance protocols.

## Figures and Tables

**Table 1:** Primers used in this study.

Gene	Primer forward (5′–3′)	Primer reverse (5′–3′)
miR-18a	5′-CGCGTAAGGTGCATCTAGTGC-3′	5′-AGTGCAGGGTCCGAGGTATT-3′
miR-34a	5′-ACACTCCAGCTGGGTGGCAGTGTCTTAG-3′	5′-TGGTGTCGTGGAGTCG-3′
miR-106b	5′-CGCACTGTGGGTACTTG-3′	5′-GTCCAGTTTTTTTTTTTTTTTGCAG-3′
miR-141	5′-ATGGTTGATGGAGCACATTG-3′	5′-CCTAACATATGAGCATGCTC-3′
miR-182	5′-TTAGGAACCCTCCTCTCTC-3′	5′-CGGTGATGTGAAGAAGGA-3′
miR-183	5′-GCGGCGGTATGGCACTGGTAGA-3′	5′-GCGGGTGCAGGGTCCGAGGT-3′
miR-200a	5′-CGGGCTAACACTGTCTGGTA-3′	5′-CAGCCACAAAAGAGCACAAT-3′
miR-200b	5′-ATCGTACGTGGGTAATACTGCCTGGTAA-3′	5′-GCAGGGTCCGAGGTATTC-3′
miR-301a	5′-GGCAGTGCAATAGTATTGT-3′	5′-TGGTGTCGTGGAGTCG-3′
miR-375	5′-AGTTTGTTCGTTCGGCTC-3′	5′-GGTCCAGTTTTTTTTTTTTTTTCAC-3′

**Table 2:** Clinical status of prostate subjects and benign prostatic hyperplasia subjects.

Items		Prostate cancer	Benign prostatic hyperplasia
Age			
	Mean	74.2	71.2
	Range	57-86	52-85
PSA			
	<2.5	1	8
	2.5-10	2	15
	10-20	2	8
	20-100	14	0
	>100	12	0
Gleason score			
	5	1	N/A
	6	2	N/A
	7	6	N/A
	8	8	N/A
	9	9	N/A
	10	5	N/A

**Table 3:** ROC analysis of miRNAs.

Predictors	ROC curves			
	Cut-off value	AUC	95% CI	*P* value
miR-141	0.708	0.894	0.820-0.969	<0.001
miR-182	1.605	0.913	0.846-0.979	<0.001
miR-200b	1.383	0.923	0.862-0.984	<0.001
miR-375	1.985	0.880	0.900-0.961	<0.001
Combinations of miR-141, miR-182, miR-200b, and miR-375	0.708	0.923	0.862-0.984	<0.001

## Data Availability

The data of this study was available at the correspondence author.
